# Effects of Smoking on Inflammatory Markers in a Healthy Population as Analyzed *via* the Gut Microbiota

**DOI:** 10.3389/fcimb.2021.633242

**Published:** 2021-07-23

**Authors:** Su Yan, Zhonghui Ma, Mengfan Jiao, Youxiang Wang, Ang Li, Suying Ding

**Affiliations:** ^1^ Health Management Center, The First Affiliated Hospital of Zhengzhou University, Zhengzhou, China; ^2^ College of Public Health, Zhengzhou University, Zhengzhou, China; ^3^ Department of Stomatology, The First Affiliated Hospital of Zhengzhou University, Zhengzhou, China; ^4^ Department of Infectious Diseases, The First Affiliated Hospital of Zhengzhou University, Zhengzhou, China; ^5^ Gene Hospital of Henan Province, The First Affiliated Hospital of Zhengzhou University, Zhengzhou, China

**Keywords:** smoking, gut microbiota, whole-genome sequencing, inflammation, healthy population

## Abstract

The number of people who smoke has increased in recent years, and the incidence of smoking-related diseases increases annually. This study was conducted to explore whether smoking affects diseases *via* changes in the gut microbiota. We enrolled 33 smokers and 121 non-smokers. We collected fecal samples from all participants and performed whole-genome sequencing. Smoking significantly affected the gut microbiota. At the phylum through genus levels, the smokers’ microbiotas showed slight changes compared with those of the non-smokers. The α- and β-diversities differed significantly between the smokers and non-smokers, and the smokers’ gut microbiota compositions differed significantly from those of the non-smokers. At the species level, the relative abundances of *Ruminococcus gnavus* (*P*=0.00197) and *Bacteroides vulgatus* (*P*=0.0468) were significantly greater in the smokers than in the non-smokers, while the relative abundances of *Faecalibacterium prausnitzii* (*P*=0.0000052) and *Akkermansia muciniphila* (*P*=0.0057) were significantly lower in the smokers. Smoking increases inflammation in the body by inducing an increased abundance of proinflammatory bacteria. Non-smokers had higher abundances of anti-inflammatory microorganisms than did smokers; these microorganisms can produce short-chain fatty acids, which inhibit inflammation.

## Introduction

Smoking is the leading preventable cause of death in humans. Smoking increases the risk of many diseases, including various cancers (Bilano et al., 2015; Jacobs et al., 2015) and cardiovascular diseases (Lubin et al., 2016). Compared with non-smokers, smokers have a 2–4-fold increased risk of coronary heart disease and stroke as well as an increased risk of atrial fibrillation (Benowitz and Burbank, 2016). Despite public awareness of the dangers of smoking, smoking rates are increasing in many developing countries (Samet and Wipfli, 2010). Smoking affects the tissues, organs and gut microbiotas in humans (Ambrose and Barua, 2004; Sasco et al., 2004; Capurso and Lahner, 2017; Huang and Shi, 2019) and alters the environment in which the gut microbes live, thereby affecting the composition and function of the gut microbiota. Cigarette smoke contains many toxic substances and may affect the microbiota by promoting cytokine production in cells, mucin production (Allais et al., 2016), changes in oxygen tension (Jensen et al., 1991), and production of reactive oxygen species in the blood (Talukder et al., 2011). It may also affect the intestinal microbiome by increasing the intestinal pH (Tomoda et al., 2011), delaying gastric emptying (Miller et al., 1989) and reducing pancreatic bicarbonate secretion (Ainsworth et al., 1993).

Previous studies have focused on the effects of smoking on the gut microbiota. One study (Lee et al., 2018; Sublette et al., 2020) showed that smokers’ gut microbiome compositions differed significantly from those of never-smokers. In those who quit smoking, the microbiome returned to a similar composition as that of never-smokers. However, another study (Shanahan et al., 2018) found significantly less diversity in the small intestinal mucosal flora of people who quit smoking than in never-smokers, implying that quitting smoking also affects microbial diversity. One study (Wang et al., 2012) showed that mice exposed to side-stream smoking exhibited significant alterations in their gut microbes, including an increased abundance of *Clostridium* and decreased Firmicutes (*Lactococcus* and *Ruminococcus*), Enterobacteriaceae and segmented filamentous bacteria as well as changes in intestinal inflammatory levels.

Because few studies have explored the influence of smoking on the gut microbiota, and most recent studies used 16S rRNA gene sequencing, the results remained at the genus level (Lee et al., 2018; Sublette et al., 2020). Here, we, for the first time, used whole-genome sequencing (WGS) to explore the effects of smoking on the gut microbiota at the species level.

## Methods

### Study Design and Sample Collection

We collected samples from the Physical Examination Department of the First Affiliated Hospital of Zhengzhou University from 2018–2019. All studies involving human participants were reviewed and approved by an ethics committee from the First Affiliated Hospital of Zhengzhou University (2018-KY-56). Subjects’ demographic information was obtained *via* standardized questionnaires. Subjects’ serological results were obtained through the hospital’s information management system. Subjects were required to have fasting blood samples drawn and stool samples collected on the same day for testing. The blood samples were sent to a clinical lab and tested using a Roche automatic biochemical analyzer Cobas-8000 (Roche, Mannheim, Germany). Inflammatory cytokines were determined using a human cytokine kit (BOSTER, Wuhan, China) and according to the manufacturer’s instructions. Fecal samples were separately packed and placed in a −80°C freezer for unified testing. Body weight, height and waist circumference were measured twice with an integrated standard. Height was measured using ultrasonic waves with an SK-X80 (Sonka, Shenzhen, China). Waist circumference was measured in the standing position at the midpoint between the lateral iliac crest and the lowest rib. Body mass index (BMI) was calculated as weight (kg) divided by height squared (m^2^). Blood pressure was measured using an Omron electronic sphygmomanometer HBP-9021 (Sonka, Shenzhen, China). Subjects were asking to rest for 10 minutes and to keep their upper limbs bare. They were measured 2–3 times, and the average was recorded.

All included subjects were healthy. Exclusion criteria were (1) any systemic disease (hypertension, diabetes, etc.); (2) excessive alcohol consumption (>25 grams/day for men and >15 grams/day for women); (3) use of any of the following drugs within the previous 6 months: antibiotics, antivirals, hypoglycemic drugs, blood pressure-lowering drugs, lipid-lowering drugs, or stomach medication; and (4) an abnormal abdominal ultrasound examination. **Figure 2A** shows the enrollment flowchart.

### DNA Extraction, Shotgun Metagenomic Sequencing and Quantity Control of Reads

DNA was extracted from 154 stool samples using the MagPure Stool DNA KF kit (Magen, China) per the manufacturer’s instructions. DNA library construction based on DNA nanospheres and shotgun metagenomic sequencing based on combined probe-anchoring synthesis were performed on all samples (MGI2000, MGI, Shenzhen, China). The overall accuracy (≥0.8) control strategy was used to perform quality control on the raw sequenced reads to filter out low-quality reads.

### Microbiome Composition and Function Profiling

Sequenced libraries were metagenomically classified using MetaPhlAn2 (Truong et al., 2015) to obtain standard relative abundance values of the species at all levels. First, the sequences and markers were compared, then the MetaPhlAn2 classifier compared the metagenomic reads against a precomputed marker catalog using nucleotide BLAST searches to provide clade abundances for one or more sequenced metagenomes. To calculate the content, the classifier normalized the total number of reads per clade by the nucleotide length of its index and provided the relative abundance of each taxonomic unit, accounting for subclade-specific indexes. Microbial clades were then estimated by normalizing read-based counts by the average genomic size of each clade. This yielded a gut microbial profile that included bacteria, archaea, eukaryotes, and viruses. The NCBI (National Center for Biotechnology Information) database (2014 edition) and the HMP Unified Metabolic Analysis Network 2 (HUMAnN2) were used to annotate the nonredundant gene sets and the functional genes into Kyoto Encyclopedia of Genes and Genomes metabolic pathways, generating the metabolic pathway compositions (Fang et al., 2018; Li et al., 2021).

### Statistical Analysis

Statistical analyses were performed using R (version 4.0.2). Standardized statistical tests were used to analyze the demographic and laboratory test results. Categorical variables are represented by counts, and chi-square tests were used for differential analyses. Continuous variables are expressed as means ± the standard deviation or medians (interquartile ranges). Between-group differences were analyzed using normality tests and homogeneity tests, where *P*>0.05 was considered normal and homogeneous. Normal and homogeneous results were analyzed using Student’s t-test or the Mann-Whitney test, respectively, where *P*<0.05 was considered statistically significant. We performed a permutational multivariate analysis of variance (PERMANOVA) and redundancy analysis (RDA) to confirm whether smoking was the most important influencing factor. We used the “vegan” package in R to calculate the Shannon, obs and Spearman indexes of each sample. Principal coordinate analysis (PCoA) was performed using the R program “ade4” for visual analysis. Differences in the microbiota at the phylum through genus levels and pathways were analyzed using STAMP (version 2.1.3). We used Welch’s t-test and multiple test correction using the Benjamini-Hochberg false discovery rate (FDR) to calculate differences between the groups. Before analyzing the differential microbiotas, we removed species with low occurrences (positivity rates <10%). We used linear discriminant analysis (LDA) effect size (LEfSe) to analyze the differences in flora compositions between groups (Segata et al., 2011). Species were displayed for LDA scores >2. Spearman correlation analysis was used to analyze the correlations between differential microbiotas and covariates, and the “corrplot” package was used for visualization.

## Results

### Participant Information

We selected 99 men and 55 women to participate in the study after they volunteered for a physical examination. Among them, 12 women and 21 men (mean age 41.67 ± 11.90 years) were defined as smokers, and 43 women and 78 men (mean age 43.03 ± 11.01 years) were defined as non-smokers. IL-10 and TNF-α differed significantly (*P*<0.05) between the smokers and non-smokers; basic body attributes, dietary habits and other inflammatory markers did not (*P*>0.05). The levels of inflammatory markers, including neutrophil counts (NEC) and monocyte counts (MOC), were slightly, but not significantly, higher in the smokers than in the non-smokers (**Table 1**).

**Table 1:** Participants’ demographic and serum characteristics.

	smoking (N=33)	non-smoking (N=121)	t/W/X^2^	*P*-value
Age^a^	41.67 ± 11.90	43.03 ± 11.01	0.667	0.506
Gender^c^	female12, male21	female43, male78	0.008	0.930
BMI(Kg/m^2^)^a^	24.53 ± 3.87	24.86 ± 3.50	0.463	0.644
Hipline(cm)^a^	81.48 ± 10.59	85.54 ± 9.99	1.787	0.077
Waist (cm)^b^	93 (88,98.5)	99 (95,102)	1072.5	0.004
Regular meals^c^	N:24, Y:9	N:109, Y:12	6.632	0.100
Dietary habit^c^	Mixed:23, meat-eating:6, vegan:4	Mixed:85, meat-eating:10, vegan:26	3.623	0.163
Wholegrains^c^	N:6, Y:27	N:22, Y:99	0.001	0.999
Yogurt^c^	N:11, Y:22	N:37, Y:84	0.092	0.762
Drinking^c^	N:9, Y:24	N:22, Y:99	0.827	0.363
Sporting^c^	not:9, rarely:13, frequently:11	not:19, rarely:59, frequently:43	2.432	0.296
SBP (mmHg)^b^	126 (119,142.5)	124.5 (114,135)	9072.5	0.457
DBP (mmHg)^a^	75.48 ± 13.21	78.90 ± 12.97	1.334	0.184
NEC(×10^9^/L)^b^	3.69 (2.53,4.35)	3.42 (2.76,4.07)	9164	0.942
LYC(×10^9^/L)^a^	2.08 ± 0.61	1.93 ± 0.50	-1.421	0.157
MOC(×10^9^/L)^b^	0.36 (0.26,0.50)	0.36 (0.30,0.46)	2380.5	0.760
WBC(×10^9^/L)^b^	6.19 (4.94,7.43)	5.90 (5.10,6.73)	9093	0.694
PLT(×10^9^/L)^a^	258.81 ± 70.45	242.68 ± 52.45	-1.432	0.154
IL-6(pg/ml) ^a^	4.59 ± 1.23	4.13 ± 0.44	-1.679	0.100
IL-8(pg/ml) ^a^	12.47 ± 9.65	10.16 ± 3.62	0.092	0.294
IL-10(pg/ml) ^a^	12.47 ± 6.07	22.99 ± 24.59	2.151	0.037
TNF-α(pg/ml) ^a^	13.80 ± 12.91	7.17 ± 6.56	-2.185	0.034
TC (mmol/L)^a^	4.73 ± 0.87	4.70 ± 0.79	-0.174	0.862
TG (mmol/L)^b^	1.06 (0.79,1.72)	1.30 (0.97,1.82)	2022	0.054
HDL (mmol/L)^b^	1.47 (1.32,1.76)	1.37 (1.17,1.58)	8777.5	0.069
LDL (mmol/L)^a^	2.83 ± 0.79	2.94 ± 0.75	0.735	0.463
FBG (mmol/L)^b^	5.04 (4.74,5.50)	5.09 (4.81,5.50)	2274.5	0.707
HbA1_c_ (%)^a^	5.65 ± 0.29	50.68 ± 0.33	0.257	0.798

BMI, body mass index; regular meals, Y = regular eating; N = irregular eating; dietary habits (mixed, meat-eating, vegan); yogurt: Y = ate yogurt every day; N = did not eat yogurt every day; drinking, Y = alcohol consumption; N = no alcohol; exercise, (no exercise, rarely exercise, frequently exercise); SBP, systolic blood pressure; DBP, diastolic blood pressure; NEC, neutrophil count; LYC, lymphocyte count; MOC, monocyte count; WBC, white blood cell count; PLT, platelet count; IL-6, interleukin- 6; IL-8, interleukin- 8; IL-10, interleukin-10; TNF-α, tumor necrosis factor α; TC, total cholesterol; TG, triglycerides; HDL, high-density lipoprotein; LDL, low-density lipoprotein; FBG, fasting blood-glucose; HbA1c, glycosylated hemoglobin. ^a^Continuous variables are presented as means (standard deviations); differences between groups were tested via Student’s t-test. ^b^Continuous but abnormal variables are presented as medians (interquartile ranges); differences between groups were tested via the Mann-Whitney test. ^c^Categorical variables are represented by counts; differences between groups were tested via the chi-square test.

### Analysis of Factors Influencing the Gut Microbes

We analyzed participants’ basic attributes (i.e., age, sex, BMI, smoking, alcohol consumption, regular diet, whole-grain consumption, yogurt consumption, exercise and inflammation increase) *via* PERMANOVA. Smoking had the greatest effect on participants’ gut microbe structure in both the univariate and multivariate analyses (*P*<0.05; **Table 2**).

**Table 2:** Influence of participants’ basic attributes on microflora structure.

Phenotype	single factor			multi-factor		
	F.Model	Variation (R^2^)	Pr (>F)	F.Model	Variation (R^2^)	Pr (>F)
Age	1.365	0.008	0.152	0.703	0.005	0.777
Gender	1.001	0.007	0.431	1.130	0.007	0.281
Regular meals	1.261	0.008	0.215	0.645	0.004	0.848
Dietary habit	1.118	0.007	0.301	1.270	0.008	0.191
Wholegrains	1.151	0.008	0.264	1.207	0.008	0.253
Yogurt	1.102	0.007	0.288	0.954	0.006	0.470
Drinking	1.606	0.011	0.077	0.804	0.005	0.655
Sporting	1.328	0.009	0.174	0.723	0.005	0.728
BMI	1.284	0.008	0.191	1.343	0.009	0.192
Inflammation	0.625	0.004	0.869	0.592	0.004	0.894
Smoking	5.987	0.038	0.001	1.923	0.013	0.035

Inflammation: Those defined as having five or more of the nine markers of inflammation above the median level of the markers.

### Differences in the Microbiota at All Levels

**Figure 1:**
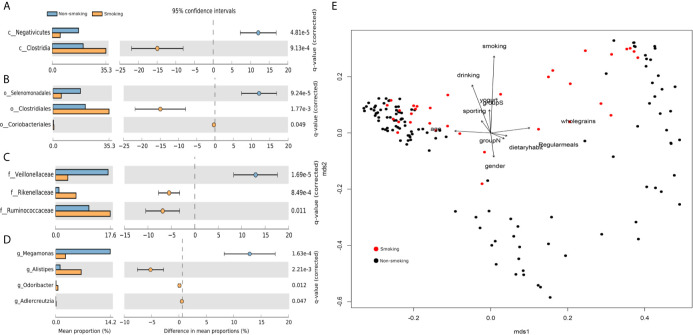
Differences at each level and RDA analysis **(A)** class-level differences; **(B)** order-level differences; **(C)** family-level differences; **(D)** genus-level differences; **(E)** effects of dietary habits and individual attributes on microflora.

We found 17 phyla, 26 classes, 50 orders, 103 families, and 265 genera. We used STAMP to calculate the differences in the microbiotas at all levels (phylum through genus). We used Welch’s t-test and multiple test correction using the Benjamini-Hochberg FDR. Two classes (**Figure 1A**), three orders (**Figure 1B**), three families (**Figure 1C**), and four genera (**Figure 1D**) differed between the smokers and non-smokers. We constructed an RDA diagram to reflect the relationship between the microflora and participants’ dietary habits and individual attributes (**Figure 1E**).

**Figure 2:**
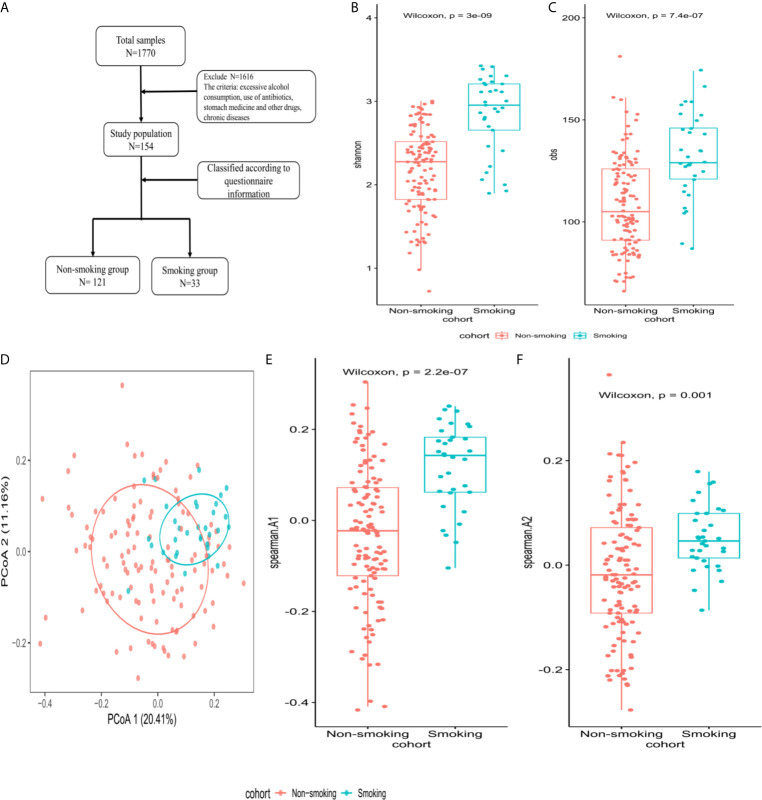
Microbiome composition and diversity **(A)** enrollment flow; **(B, C)** Shannon and obs indexes were used to analyze α-diversity between smokers (N=33) and non-smokers (N=121); **(D)** β-diversity using PCoA diagrams of the Spearman distance; **(E, F)** differences in the first and second principal components between smokers and non-smokers by Spearman distance.

The Shannon and obs indexes showed that α-diversity differed significantly between smokers and non-smokers (*P*<0.001, **Figures 2B, C**). Spearman analysis of the β-diversity showed that the smokers and non-smokers were well separated (**Figure 2D**). The Spearman distance showed significant differences in the first and second principal components between the smokers and non-smokers (*P*<0.001; **Figures 2E, F**).

### Differences and Correlation Analysis of the Microbiotas at the Species Level

**Figure 3:**
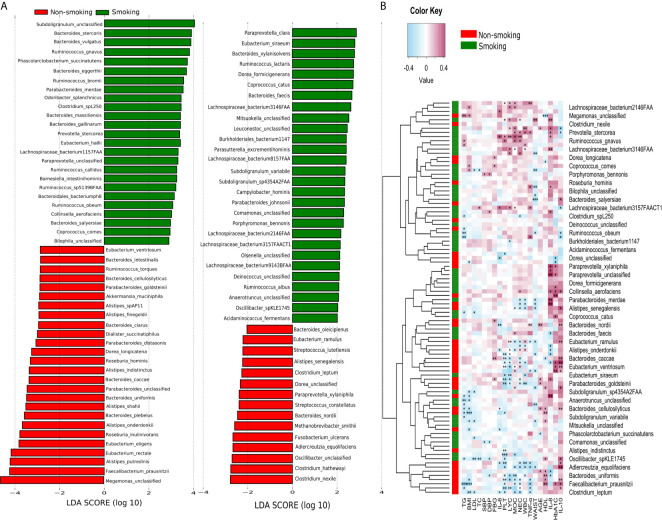
LEfSe and Spearman correlation analysis **(A)** LEfSe analysis shows microbial species with LDA scores >2; **(B)** Spearman correlation analysis showing the correlations between species abundances and participant characteristics. ^*^ *P* < 0.05, ^**^ *P* < 0.01, ^***^ *P* < 0.001.

LEfSe analysis revealed that 94 species differed significantly between the smokers and non-smokers. Fifty-three species were enriched in the smokers, including *Bacteroidales bacterium pH8*, *eggerthii*, *faecis*, *gallinarum*, *massiliensis*, *salyersiae*, *stercoris*, *vulgatus* and *xylanisolvens*; *Lachnospira bacterium1157FAA*, *bacterium2146FAA*, *bacterium3146FAA*, *bacterium3157FAACT1*, *bacterium8157FAA* and *bacterium9143BFAA*; and *Ruminococcus albus*, *bromii*, *callidus*, *gnavus*, *lactaris*, *obeum* and *sp5139BFAA*. Forty-one species were enriched in the non-smokers, including *Alistipes finegoldii*, *indistinctus*, *onderdonkii*, *putredinis*, *senegalensis*, *shahii* and *spAP11*; *Bacteroides caccae*, *cellulosilyticus*, *clarus*, *intestinalis*, *nordii*, *oleiciplenus*, *plebeius* and *uniformis*; *Eubacterium eligens*, *ramulus*, *rectale* and *ventriosum*; and *Roseburia hominis*, *torques* and *inulinivorans* (LDA>2, **Figure 3A**). **Supplementary Table 1** shows the detailed microbiota results. Spearman’s correlation analysis was used to explore the correlations between species abundances and participant characteristics (**Figure 3B**). Some bacteria, including *Bacteroidales bacterium pH8*, *B. eggerthii*, *Ruminococcus albus*, and R*. bromii*, were positively correlated with inflammatory markers (i.e., NEC, LYC, MOC, WBC, and PLT) and were enriched in the smokers. Other bacteria, such as *Eubacterium eligens*, *ramulus*, *rectale* and *ventriosum* and *Roseburia hominis*, *torques* and *inulinivorans* were negatively correlated with inflammatory markers and were enriched in non-smokers.

### Functional Shifts in Participants’ Microbiome Characteristics

**Figure 4:**
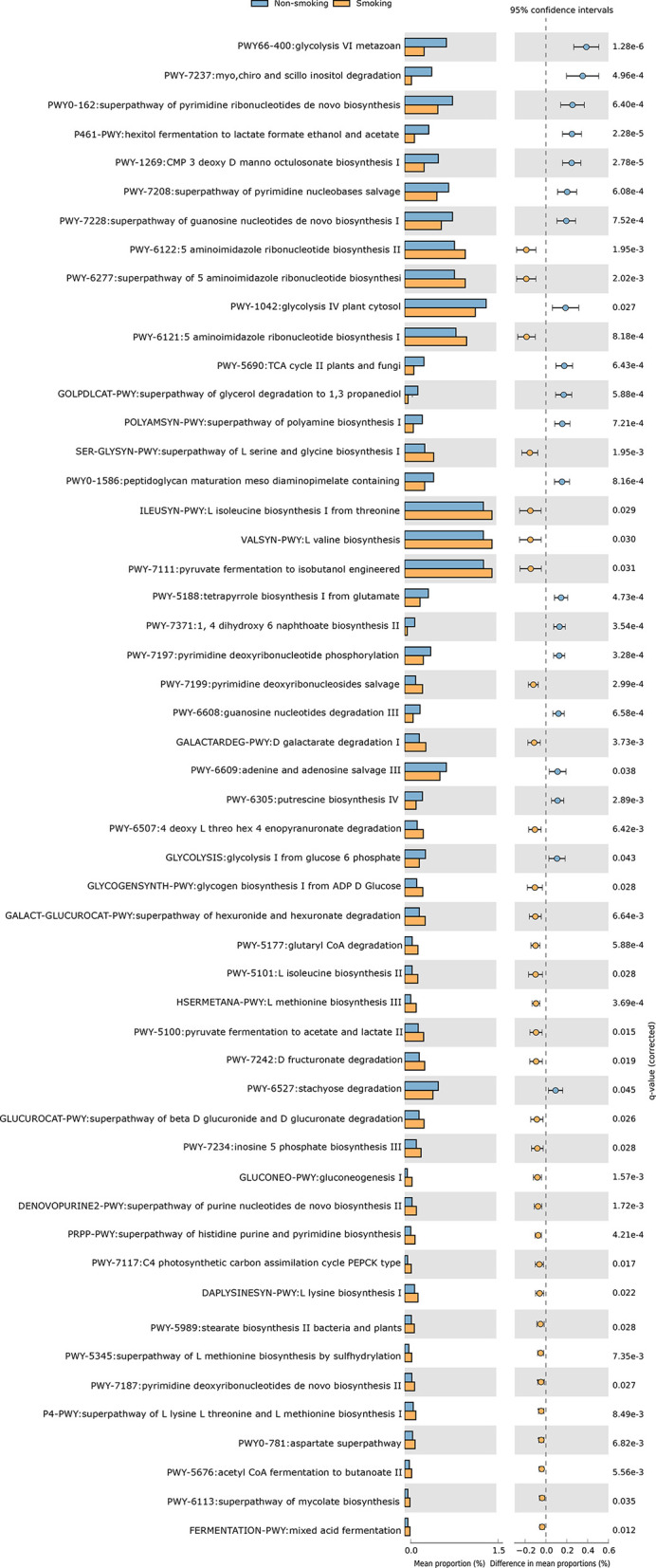
Functional shifts in bacterial species between smokers and non-smokers. Fifty-two pathways differed significantly, and 32 were enriched in smokers (*P*_corrected_ *< *0.05).

We constructed functional profiles for each sample using 494 microbial MetaCyc pathways. After removing the low-abundance pathways, 52 pathways differed significantly between the smokers and non-smokers (**Figure 4**), and 32 of these were enriched in the smokers. Within these 32 pathways, nine were responsible for amino acid synthesis (SER-GLYSYN-PWY, ILEUSYN-PWY, VALSYN-PWY, PWY-5101, HSERMETANA-PWY, DAPLYSINESYN-PWY, PWY-5345, P4-PWY, and PWY0-781); two were responsible for degrading amino acids and nucleotides (PWY-5100 and GALACTARDEG-PWY); six were responsible for nucleoside synthesis (PWY-6122, PWY-6277, PWY-6121, PWY-7199, DENOVOPURINE2-PWY, and PRPP-PWY); two were responsible for generating precursor metabolites and energy (PWY-7117 and PWY-5676); seven were responsible for biosynthesis (PWY-5177, PWY-7234, PWY-5989, PWY-7187, PWY-6113, GLYCOGENSYNTH-PWY, and GLUCONEO-PWY), and six were responsible for degrading carbohydrates (PWY-7111, PWY-6507, GALACT-GLUCUROCAT-PWY, PWY-7242, GALACT-GLUCUROCAT-PWY, and FERMENTATION-PWY). Of the pathways enriched in the non-smokers, five were responsible for nucleoside synthesis (PWY0-162, PWY-7208, PWY-7228, PWY-7197, and PWY-6609), six were responsible for biosynthesis (PWY-1269, PWY0-1586, PWY-5188, POLYAMSYN-PWY, PWY-7371, and PWY-6305), four were responsible for carbohydrate degradation (PWY66-400, P461-PWY, PWY-1042, and GOLPDLCAT-PWY), three were responsible for degrading amino acids and nucleotides (PWY-7237, PWY-6608, and PWY-6527), and two were responsible for generating precursor metabolites and energy (PWY-5690 and GLYCOLYSIS).

## Discussion

**Figure 5:**
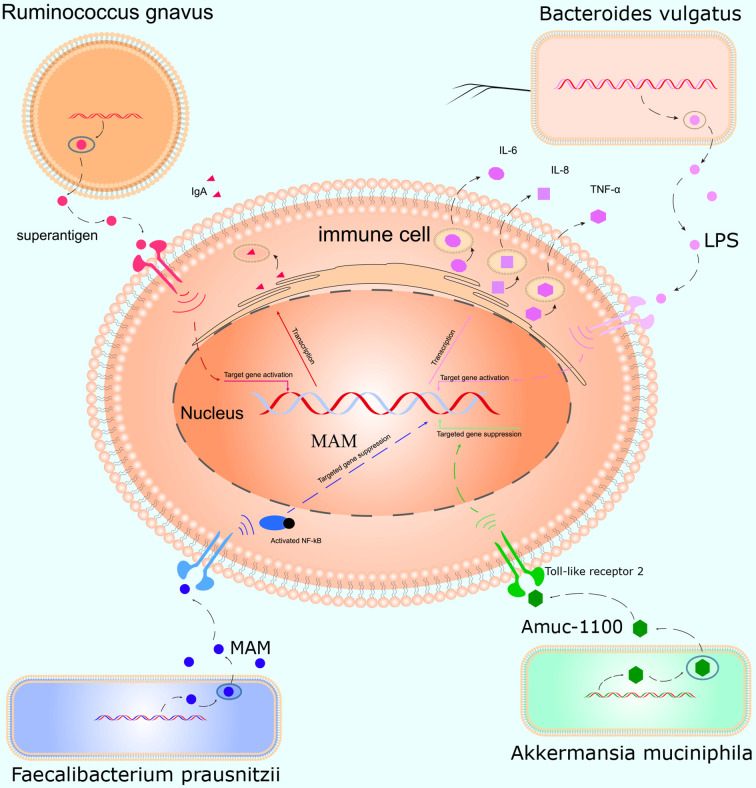
Mechanism of action of the gut microbiota in inflammation. *Ruminococcus gnavus* contains specific genes that encode superantigens to induce and bind IgA antibody *in vivo*. *Bacteroides vulgatus* produces LPS, which activates T cells and promotes immune cells to produce cytokines such as IL-6, IL-8, and TNF-α. *Faecalibacterium prausnitzii* inhibits inflammation by producing MAM to inhibit NF-κB activation in the intestinal microbiome. *Akkermansia muciniphila* secretes the peptide, Amuc_1100, which interacts with Toll-like receptor 2, improves the gut barrier and decreases inflammation.

This study was conducted to initially explore the effects of smoking on the gut microbiota and understand how smoking affects the body. We, for the first time, used WGS to detect the effects of smoking on the gut microbiota. We found that smoking significantly affected the gut microbiota, and the α- and β-diversities differed significantly. At the species level, compared with those of non-smokers, the relative abundances of *Bacteroidales bacterium pH8*, *eggerthii*, *faecis*, *gallinarum*, *massiliensis*, *salyersiae*, *stercoris*, *vulgatus* and *xylanisolvens*; *Lachnospira bacterium1157FAA*, *bacterium2146FAA*, *bacterium3146FAA*, *bacterium3157FAACT1*, *bacterium8157FAA* and *bacterium9143BFAA*; and *Ruminococcus albus*, *bromii*, *callidus*, *gnavus*, *lactaris*, *obeum* and *sp5139BFAA* increased, while the relative abundances of *Alistipes finegoldii*, *indistinctus*, *onderdonkii*, *putredinis*, *senegalensis*, *shahii* and *spAP11*; *Bacteroides caccae*, *cellulosilyticus*, *clarus*, *intestinalis*, *nordii*, *oleiciplenus*, *plebeius* and *uniformis*; *Eubacterium eligens*, *ramulus*, *rectale* and *ventriosum*; and *Roseburia hominis*, *torques* and *inulinivorans* decreased in the smokers. Interestingly, the gut microbes that were enriched in the smokers were positively correlated with inflammatory markers, and the gut microbes that were enriched in the non-smokers were protective factors and were negatively correlated with inflammatory markers (**Figure 5**).

From the known effects of smoking, including immune system alterations (Sorensen et al., 2010; Mahassni and Alajlany, 2020), direct antibacterial activity (Pavia et al., 2000) and changes in oxygen tension (Jensen et al., 1991), we can propose many hypotheses related to our findings and to the observed changes in bacterial community compositions. For non-smokers, α- and β-diversities in the gut microbiota differed significantly from those of smokers. Microbiota diversity is generally associated with health (Requena et al., 2018), and changes in immune homeostasis and reduced diversity due to smoking may adversely affect the disease statuses of smokers relative to microbial-immune interactions; thus, further study of these interactions is warranted.

*Eubacterium ramulus*, *E. rectale* and *E. ventriosum* were enriched in the non-smokers and were the bacteria most negatively correlated with inflammatory markers and produced large amounts of short-chain fatty acids (SCFAs). *E. ramulus*, *E. rectale* and *E. ventriosum* digested polysaccharides to produce SCFAs (Barcenilla et al., 2000; Pryde et al., 2002; Macfarlane and Macfarlane, 2011), which play key protective roles against inflammation. *E. eligens* strongly promoted production of IL-10, an anti-inflammatory cytokine, in *in vitro* cell experiments (Chung et al., 2017). These findings are consistent with our finding that these species were negatively correlated with inflammatory indicators. The next most relevant bacterium was *Adlercreutzia equolifaciens*, which was enriched in the non-smokers; it converts ingested isoflavones, which are abundant in legumes and soya beans, into equol (Maruo et al., 2008). Equol has a high affinity for the estrogen receptor (Setchell et al., 2009) and may be a selective estrogen receptor modulator. *A. equolifaciens* is involved in metabolizing polyphenols and produced bioactive molecules involved in ameliorating metabolic disorders in obesity and diabetes (Clavel et al., 2014). *Akkermansia muciniphila* is also strongly correlated with inflammatory markers. *A. muciniphila* adheres to the mucosal layer, may have beneficial properties (Zhang et al., 2019), and increases intestinal endocannabinoids, which control inflammation (Plovier et al., 2017) and have potential anti-inflammatory properties (Derrien et al., 2017). Additionally, Amuc_1100 (Plovier et al., 2017), a protein isolated from *A. muciniphila*, interacts with Toll-like receptor 2 and helps improve the gut barrier. *Alistipes finegoldii, indistinctus*, *onderdonkii*, *putredinis*, *senegalensis*, *shahii* and *spAP11* were enriched in non-smokers and are reported to reduce gut inflammation. This is consistent with our findings, in which these species were negatively correlated with inflammatory markers.

*Bacteroides caccae, cellulosilyticus, clarus, intestinalis, nordii, oleiciplenus, plebeius* and *uniformis* were enriched in non-smokers. *B. caccae, B. intestinalis* and *B. uniformis* significantly reduced the release of lipopolysaccharide-induced IL-8 from HT-29 cells (Hiippala et al., 2020). In fecal microbiota transplantation, increased *B. plebeius* was associated with disease in patients with colitis. *Clostridium leptum* increased regulatory T cells in the spleen (Li et al., 2012) and inhibited inflammatory cytokine production to decrease inflammation (He et al., 2020) in mice. *Faecalibacterium prausnitzii* secreted seven peptides belonging to the microbial anti-inflammatory molecule (MAM), which inhibited the NF-κB pathway *in vitro. F. prausnitzii* also protects against inflammation in the gut by producing butyrate, an SCFA (Sokol et al., 2008; Ganesan et al., 2018). *Parabacteroides distasonis, goldsteinii, unclassified* and *xylaniphila* were enriched in non-smokers and helped decrease inflammation. P. distasonis reduced the expressions of Toll-like receptor 4, IL-4 and TNF-α and increased IL-10 expression in the colon (Koh et al., 2020). *P. distasonis* inhibits TNF-α production *via* macrophages *in vitro* (Kverka et al., 2011). *P. goldsteinii* can reduce intestinal inflammation and inhibit lung inflammation, and lipopolysaccharides derived from *P. goldsteinii* are anti-inflammatory (Lai et al., 2021). *Roseburia hominis* and *inulinivorans* were enriched in non-smokers. These bacteria all produce butyrate and SCFAs, which break down polysaccharides and reduce inflammation (Chu et al., 2019; Ticinesi et al., 2020; Zheng et al., 2020).

*Bacteroides vulgatus* and *B. xylanisolvens* were enriched in the smokers and were the most positively correlated with inflammatory markers. *B. vulgatus* and *B. xylanisolvens* are considered to promote colitis (Dziarski et al., 2016). *B. vulgatus* and *B. xylanisolvens* are Gram-negative and trigger systemic inflammation, including increased IL-6 and THF-α and insulin resistance (Leite et al., 2017; Higuchi et al., 2018). *B. vulgatus* stimulates CD4 cells and secretes specific antigens to induce colitis (Hoentjen et al., 2007; Kathania et al., 2020). *Lachnospira bacterium1157FAA*, *bacterium2146FAA*, *bacterium3146FAA*, *bacterium3157FAACT1*, *bacterium8157FAA* and *bacterium9143BFAA* were enriched in the smokers and were positively correlated with inflammatory markers. One study found that a high-fat diet increased the abundance of *Lachnospira* and the inflammatory status in mice with colitis (Zeng et al., 2016). *Prevotella stercorea* was positively correlated with colonic dendritic cell activation levels *in vivo* and increased strong proinflammatory cytokine production. Consistent with our findings, *Ruminococcus albus*, *bromii*, *callidus*, *gnavus*, *lactaris*, *obeum* and *sp5139BFAA* promoted inflammation. *R. gnavus* can produce specific antigens and stimulate immune cells to produce corresponding antibodies, thus increasing inflammation (Hall et al., 2017; Bunker et al., 2019).

Although the metabolic pathways in the gut microbiotas differed between the groups, these differences were not functionally significant. In both groups, several pathways, whose functions included nucleoside synthesis, biosynthesis, degradation of carbohydrates, degradation of amino acids and nucleotides, and generation of precursor metabolites and energy, were individually enriched. Interestingly, nine distinct pathways that were enriched in smokers were responsible for amino acid biosynthesis, including L-serine, glycine, L-isoleucine, L-valine, L-methionine, L-lysine, L-threonine and aspartate. Our hypothesis is that accumulation of amino acids, the basic structures of proteins, may be due to the increased demand for amino acids caused by changes in the bacterial abundances due to smoking, thereby increasing the amino acid abundances.

The participants in our study came in for routine checkups and were thus more likely to represent the healthy population in China. We implemented a rigorous screening process and excluded those with systemic diseases, excessive alcohol consumption, drug use and enteric diseases, which was an advantage of this study. Our analysis included both smokers and non-smokers. However, participants were more likely to underestimate their smoking habits, resulting in their actual smoking status being unreported. Participants’ daily environments were also unknown (e.g., passive smoking) and may have influenced our results, which may have influenced our analysis. Therefore, these factors must be further analyzed to understand the impact of smoking on the gut microbiota.

## Conclusion

Smoking alters bacterial communities in the stool, and this alteration may be associated with inflammation. Many studies have shown that the main characteristic of chronic diseases is systemic inflammation. Smoking increases inflammation in the body by causing accumulation of inflammation-promoting bacteria, such as *Bacteroides*, *Lachnospira*, *Prevotella stercorea* and *Ruminococcus*, in the gut. Conversely, the microbiotas of non-smokers were enriched with bacteria that inhibit inflammation, including *Eubacterium*, *Adlercreutzia equolifaciens*, *Akkermansia muciniphila*, *Alistipes* and *Bacteroides*. The mechanism of interaction between the gut microbiota and inflammation requires further study.

## Data Availability Statement

The datasets presented in this study can be found in online repositories. The names of the repository/repositories and accession number(s) can be found below: https://www.ebi.ac.uk/ena, PRJEB36271.

## Ethics Statement

The studies involving human participants were reviewed and approved by Ethics Review Committee of scientific Research Projects in the First Affiliated Hospital of Zhengzhou University. The patients/participants provided their written informed consent to participate in this study. Written informed consent was obtained from the individual(s) for the publication of any potentially identifiable images or data included in this article.

## Author Contributions

SY researched the data, performed the analysis and wrote and edited the manuscript. ZM and MJ helped to collect the data and edit the manuscript. AL is the guarantor of this work and as such had full access to all data in the study and takes responsibility of the integrity of the data and the accuracy of the data analysis. SD was in charge of the project. All authors contributed to the article and approved the submitted version.

## Funding

This research was equally funded and supported by the Chinese National Science and Technology Major Project (2018ZX10305410), the Henan Province Medical Science and Technique Project grant (2018020001), the Henan Province Postdoctoral Research grant (001801005) and the Key Scientific Research Projects of Universities in Henan Province (21A320035).

## Conflict of Interest

The authors declare that the research was conducted in the absence of any commercial or financial relationships that could be construed as a potential conflict of interest.

## Publisher’s Note

All claims expressed in this article are solely those of the authors and do not necessarily represent those of their affiliated organizations, or those of the publisher, the editors and the reviewers. Any product that may be evaluated in this article, or claim that may be made by its manufacturer, is not guaranteed or endorsed by the publisher.
